# Double threshold in bi- and multilingual contexts: preconditions for higher academic attainment in English as an additional language

**DOI:** 10.3389/fpsyg.2014.00546

**Published:** 2014-06-05

**Authors:** Simone Lechner, Peter Siemund

**Affiliations:** Department of English and American Studies, University of HamburgHamburg, Germany

**Keywords:** attainment of academic literacy, bilingualism, English as a foreign language, English as an additional language, migrant languages, third language acquisition, threshold hypothesis

## Abstract

Bi- and multilingualism has been shown to have positive effects on the attainment of third and additional languages. These effects, however, depend on the type of bi- and multilingualism and the status of the languages involved (Cenoz, 2003; Jessner, 2006). In this exploratory trend study, we revisit Cummins' Threshold Hypothesis (1979), claiming that bilingual children must reach certain levels of attainment in order to (a) avoid academic deficits and (b) allow bilingualism to have a positive effect on their cognitive development and academic attainment. To this end, we examine the attainment of English as an academic language of 16-years-old school children from Hamburg (*n* = 52). Our findings support the existence of thresholds for literacy attainment. We argue that language external factors may override positive effects of bilingualism. In addition, these factors may compensate negative effects attributable to low literacy attainment in German and the heritage languages. We also show that low attainment levels in migrant children's heritage languages preempt high literacy attainment in additional languages.

## Introduction

In Germany and elsewhere, it remains highly debated if bilingualism has a positive impact on cognitive development or academic attainment (Gogolin and Neumann, 2009), especially in subtractive bilingual situations (Cenoz, 2003, pp 74–80). Several studies have shown that bilinguals have cognitive advantages compared to their monolingual peers (Bialystok, 2009, pp 97–98), particularly in tasks that require cognitive flexibility and selective attention (Bialystok et al., 2009, p. 230). At the same time, however, bilingual children have been shown to be disadvantaged as far as lexical retrieval is concerned (Bialystok, 2009, p. 55).

Regarding the attainment of additional languages, positive effects have been identified for bilingual learners in comparison to their monolingual peers (Cummins, 1992, p. 65; Jessner, 2006, p. 27). Such positive effects seem to manifest themselves only once certain attainment levels have been reached. Swain et al. (1990 p. 73) claim that bilingual literacy development is especially important in this respect. Furthermore, language external variables seem to play an important role (De Angelis, 2007, p. 12).

According to the German DESI-study (DESI-Konsortium, 2006), subtractive bilingual heritage speakers show slight advantages in the attainment of third or additional languages, interpreted by Burghardt and Esser (2008), Esser (2009) as an effect of learning German as a home language, rather than an effect of bilingualism itself. Overall, research on third language literacy attainment has led to mixed results concerning effects attributable to subtractive bilingualism (Cenoz, 2003, p. 83).

Cummins' Threshold Hypothesis (1979, p. 227) provides an explanation of different academic outcomes in subtractive bilingual situations. Cummins here postulates that bilingual children have to reach a certain level of attainment in both their L1 and their L2 for positive effects of bilingualism to play out.

Cummins' Threshold Hypothesis has been the subject of much criticism and debate, particularly with regard to the notion of “limited bilingualism” (MacSwan, 2000, p. 5). It has been argued that limited bilingualism carries pejorative connotations in the same way as the term “semilingualism” does. Consequently, researchers who use the term “limited bilingualism” view certain types of bilingualism from a deficit perspective (MacSwan and Rolstad, 2006, p. 2309).

We believe that it is necessary to distinguish between linguistic competence and performance (langue versus parole) in the interpretation of the Threshold Hypothesis. Cummins seems to use the term “language proficiency” to refer to both competence and performance, including school literacy. MacSwan (2000, pp 33–34) argues that if the Threshold Hypothesis refers to language competence, it is spurious because there is no evidence to suggest that subtractive bilinguals did not know the underlying principles of their language. If, on the other hand, the Threshold Hypothesis refers to school literacy, it is irrelevant and tautological (MacSwan, 2000, p. 34), because in this case literacy and related school knowledge would have to be viewed as aspects of language itself rather than academic achievement. Following MacSwan (2000), we here view limited bilingualism or semilingualism as completely unrelated to linguistic competence. If at all, these notions say something about the failure to perform according to certain cultural norms.

We do not regard the Threshold Hypothesis as a competence-related construct, but rather as a performance-based concept relating to educational attainment. Performance data are used to measure attainment, which only reflect the underlying linguistic competence. In this descriptive trend study, we investigate the effects of high and low attainment levels in informants' heritage language and their language of environment, i.e., German, on their attainment of English as a foreign language. We aim to do so by investigating the attainment of academic literacy in English by Turkish-German, Vietnamese-German, and Russian-German simultaneous and successive bilinguals in subtractive bilingual contexts who acquire English as their third or additional language.

The present study aims to investigate whether 16-years-olds with high literacy assessment scores in English also achieve high literacy assessment scores in German and their heritage languages, and whether a high assessment score in the heritage language in addition to the L2 German has a perceivable positive influence on literacy in the target language English.

It is important to emphasize that we are here measuring aspects of language performance, especially literacy achievement, and not proficiency understood as competence. To avoid confusion, we here opt to avoid the term “proficiency” altogether and use the terms “attainment” and “literacy achievement” instead. Furthermore, we here define the attainment of English as an academic achievement, i.e., educational attainment. We argue that the effects we can observe depend on a double threshold, meaning that thresholds for positive influence of literacy skills in informants' background languages are lower if socioeconomic factors are favorable.

## Methods and data

The data presented here are the result of a panel study conducted in the context of the research cluster *Linguistic Diversity Management in Urban Areas* (LiMA-LiPS, 2009–2013), more specifically a pilot for a panel study initiated in the cluster that is still ongoing. Panel studies are defined as longitudinal studies that measure the same variables on the same units, in our case informants, over time. They therefore consist of multiple waves of testing. Two waves of testing were completed in the context of the LiPS pilot study. The panel study investigates the development of heritage languages and the language of environment (i.e., German) for children (aged 6, 11, 15 in the first wave of testing). They come from different migrant communities (Russian-German, Turkish-German, and Vietnamese-German) and a German control group, living in the urban space of Hamburg. In total, the LiPS pilot study tested 150 informants in each language group distributed equally across the three age groups.

We extracted *n* = 132 informants from the two older age cohorts (aged 12 and 16 at the time of data collection) in the second wave of this pilot study (henceforth *main panel*). In this study, we will only be focusing on the 16-years-old informants. The informants represent each of the language groups. The initial target number of participants we aimed to extract from the main panel was *n* = 160 (*n* = 40 for each language group, with *n* = 20 12-years-old and *n* = 20 16-years-old), but we encountered difficulties extracting data from the Turkish-German group in particular. In cases where the target number could not be achieved, additional interviews were conducted. Furthermore, we conducted interviews with monolingual student control groups equally distributed across the same ages with English as their L2 in Russia, Turkey, and Vietnam.

We differentiate between bilingual and monolingual informants, although it is possible to construe our German control group as bilinguals and our bilingual informants as multilinguals, as both groups have acquired English in addition to their native languages. We here opt to use the terms “bilingual” and “monolingual” because these appear in the main panel. Furthermore, we use the term “heritage language” instead of “home language” or “community language,” because it is the term utilized in the main panel.

Background variables and informants' attainment levels for different text types, both in German and their heritage language, were tested in the context of the main panel study. When additional interviews were conducted, we relied on questionnaires with smaller sets of background variables. In addition to this, we conducted an additional socioeconomic background interview and a parental questionnaire with all of our informants.

The English language tasks consisted of an oral description based on a picture sequence, a written narrative based on a picture sequence, and an academic language task, likewise based on a sequence of pictures. The latter was only conducted with the 16-years-old and aimed for instructive texts. All of the instruments were piloted in advance with children of the same ages and with the same language backgrounds as well as with adults. We piloted specifically for manageability of the task to avoid any cultural or sex-based bias. Interviews were conducted by bilingual interviewers in the children's homes. Most of the bilingual children we interviewed spoke primarily their heritage language at home, while the German monolinguals spoke only German at home. The informants were chosen based on a mixed method of random sampling relying on data obtained from Hamburg's registration office and snowballing. The aim was to collect representative data rather than data from homogeneous groups, as we have informants with a wide range of socioeconomic and educational backgrounds in our sample.

The instructive task was an English translation of the first part of *Fast Catch Bumerang*,^1^ a task which had been developed in the context of FÖRMIG (Reich et al., 2009). In the context of the main panel study, it had been conducted in German and informants' heritage languages with the oldest age group. The task had previously been used in large-scale projects in different languages, and was completed in English for the first time in the context of the LiPS pilot study (and therefore underwent additional piloting). It is an instrument aimed at measuring academic language that is not based on curricular goals. The instrument contains a set of six pictures showing the construction of a boomerang. The task is to write an instructive text describing the construction of the boomerang, understandable without the pictures. We allowed 20 min to complete this task. Two native speakers of English scored the task independently, thus increasing interrater reliability.

Six-digit numbers were assigned to each of the informants as IDs. The first digit encodes for city (1 = Hamburg; 2 = Lüneburg), the second digit encodes for language group (1 = L1 Russian, 2 = L1 Turkish, 3 = L1 Vietnamese and 4 = L1 German), and the third digit encodes for the age of the informants (2 = 12-years-old, 3 = 16-years-old). The last three digits are randomized sequences that allow us to identify each informant and to assign background variables from different testing sequences to each of them.

In this trend study, we focus exclusively on 16-years-old informants who were also part of the main panel and handed in completed versions of the Boomerang task in English,^2^ because these informants completed this task in all three languages, i.e., English, German, and their respective heritage language. This leaves us with *n* = 52 informants, distributed as follows: *N* = 20 Russian-German bilinguals, *n* = 11 Vietnamese-German bilinguals, *n* = 5 Turkish-German bilinguals, and *n* = 16 German monolinguals.

In a first step, we will look at all of the informants and take into account not only the language assessment scores for German, their heritage languages, and English, but also socioeconomic background variables that have been shown to have effects on language development and, subsequently, on language production. We try to assess whether bilinguals with similar socioeconomic backgrounds show differences in comparison to their monolingual peers regarding their production of instructive texts in English. In a second step, we will look at the *n* = 10 informants with the highest and lowest proficiency scores in English, and examine whether there are any correlations between language assessment scores in German and/or the heritage languages.

To this end, we will compare their scores in English to their scores in German and their heritage languages, as supplied by the main panel. It needs to be pointed out that different scoring schemes were used in the main panel and our study. These differences in measurement are due to the fact that students encounter English almost exclusively as a school subject, while they encounter both German and their heritage languages in informal environments. While the target varieties for German and children's heritage languages are diverse, there is a clear target variety for English, i.e., standardized British or American English, as it is taught in the German school context. Moreover, informants often have experienced no formal language education in their heritage languages. Consequently, while an overall correctness score is part of the English scoring paradigm, it is not part of the scoring paradigms for either German or the heritage languages.

## Preliminary results

### Typologies of the languages involved

The typologies of the background languages of our informants are highly diverse. Russian is an inflectional-fusional language that has free word order (but a preference for SVO). It has no articles and fairly complex conjugation and declension paradigms (Wade, 2011). Vietnamese is a tonal, isolating language with an SVO word order (Ngô, 2001). Although it has a complex classifier system, there are no word classes that correspond directly to articles. Turkish is an agglutinating language with basic SOV word order (Göksel and Kerslake, 2011). It has multiple ways of expressing indefiniteness of the noun phrase, including an indefinite article. There is no definite article, though definiteness may be marked via declension.

German, the language of environment for all of the informants in our sample, has V2 word order in main clauses and SOV patterns in subordinate clauses. It has indefinite and definite articles and is classified as a moderately inflecting language. English, finally, is a weakly inflecting language. It has a basic SVO word order and definite as well as indefinite articles.

Based on the typologies of the languages involved, positive as well as negative transfer to the target language English is hypothetically possible from all of the source languages, i.e., informants' heritage languages and the language of environment, German. We here define transfer in the sense of Odlin's (1989) “cross-linguistic influence” and view it as a bilateral process with possible facilitation and interference outcomes. Due to the bi- and multilateral nature of transfer phenomena, it is therefore also possible for English to have an influence on German and the heritage languages, although this will play no role in the study presented here.

### Scores in english with regard to school type

Scores for each of the tasks in English were measured as a combined score of lexical richness (types/tokens, lemmas/tokens), structural complexity (number of subordinating and coordinating conjunctions, clauses, relative clauses, sentences and passives in relation to token output), overall correctness score (target-like occurrences/tokens), and the length of the text. The score for the length of the text was measured against the highest token output, i.e., the text with the highest number of produced words, in the sub-sample. This method led to scores that range from 8 to 70, with the majority ranging from 40 to 70. In principle, it would have been possible to achieve higher scores, but none of the informants in our subsample achieved a score higher than 70. In a next step, scores were transferred to a categorical scoring system that ranges from zero to seven (zero being the lowest and seven being the highest), which in turn corresponds to a category in the Common European Framework of References for Language (henceforth CEFR; Little, 2006). These categories are based on the Can-Do-Statements issued by the European Union. The highest possible score in the categorical scoring system corresponds to the C2 category in the CEFR. None of the informants in our subsample reached a C2 level, six being the highest score achieved. The categorical scores were established by three independent raters based on the scores achieved in the written task. Individuals with comparatively lower scores out of 70 were able to achieve comparatively higher CEFR scores if their writing corresponded to a higher CEFR level according to the Can-Do-Statements. Because our scores included lexical richness and structural complexity, however, informants with lower total scores did not achieve higher scores than informants with higher scores in the categorical scoring system.

The three different categories are illustrated for informant 143032 (German monolingual) in Table 1.

**Table 1:** **Categorization of attainment scores**.

**ID**	**Total score**	**Total score English**	**CEFR**
143032	68.60	6.0	C1

The scores of all *n* = 52 informants investigated for the purposes of this study are listed from highest to lowest in Supplementary Table 1 (see Supplementary Material). They were generated for the Boomerang task. We generated independent scores for the other tasks. These may differ from results in the Boomerang task, as they measure other aspects of language production. In Supplementary Table 1, informants are listed according to their ID and language group. In addition to the scores, the following information is given: language background, informants' sex, age of onset for German, the HISCED^3^ index (highest educational background in informants' families), the HISEI^4^ index (highest socioeconomic background of the informants' households), and school type. The 6-digit ID serves as a reference point for the background data and the scores.

All of the bilingual informants in our subsample save one (133044) used their heritage language as their primary language of communication with their parents and spoke primarily their heritage language at home. Some of the Russian-German bilinguals (those who had started acquiring German after the age of six) underwent schooling and literacy development in Russian.

Germany has a tracked school system. The *Gymnasium* is the highest form of secondary schooling. The biggest gaps in attainment levels can normally be observed between students who attend the *Gymnasium* and those attending other school forms. Differences in attainment between other school types are not as high. The *Stadtteilschule*—like the *Gesamtschule*—is a hybrid combining middle and secondary school tracks. The *Realschule* offers middle school education only. There are special school forms, such as the *Förder-* and *Sonderschule*, which tend to children with learning disabilities and are attended by students with significantly lower attainment levels. Differences in attainment within classes of the same school type are typically not as high in countries with tracked school systems as they are in countries with comprehensive school systems (Dronkers et al., 2011, p. 31).

The school type informants attend can be viewed as a marker of their educational attainment, but it is important to keep in mind that school type is a symptom of multiple underlying variables. For informants who attend higher secondary education, these variables can normally be explained in terms of favorable socioeconomic conditions and high educational backgrounds in their families. There are exceptions, though, as evidenced in Supplementary Table 1.

The majority of informants achieving high scores in the English Boomerang task attends the *Gymnasium* and comes from families with high educational backgrounds. The two informants with the lowest overall attainment scores for the Boomerang task are 143565 and 143327 (see Supplementary Table 1), both German monolinguals, who attend schools for children with learning disabilities. They completed the task primarily in German with interspersed English function words. The informants at the lower end of the scoring spectrum attend diverse school forms. Most of the informants who do not attend the *Gymnasium* achieve comparatively lower scores. In cases in which informants attend the *Gymnasium* and still have lower scores for English, we typically find comparatively lower HISEI and HISCED scores. The educational background of informants' families seems to play a more pronounced role than household income. Age of onset seems to be irrelevant, as we find simultaneous and successive bilinguals both at the higher and lower end of the scoring spectrum. Similarly, sex does not seem to be an influencing factor.

Although influences from the heritage languages are observable, especially with informants who acquired German after the age of three, the majority of non-target-like effects occur regardless of differences in language background. They may be explainable in terms of (partly fossilized) language acquisition stages.

Non-target-like occurrences in informants' texts include code mixing, as in Example 1. It occurs exclusively from German, especially at the lower end of the scoring spectrum. Moreover, we find non-target-like subject-verb-agreement and tense- and aspect marking, again primarily at the lower end of the scoring spectrum. This is illustrated in Example 2. Non-target-like word order, particularly in the placement of adverbs and prepositional phrases, is portrayed in Example 3. The latter phenomenon occurred regardless of the scores that informants' achieved. As most informants were not accustomed to the specialized English vocabulary the task required, all of them used coping strategies such as direct lexical transfer (Example 4) and paraphrasing (Example 5). We use angled brackets to indicate the non-target like specimens under discussion.

1. 123241 Turkish-German bilingual
           I have a Boomerang <Schablone ‘template’>, we <schneiden es ‘cut it’>
2. 133150 Vietnamese-German bilingual
           […] that you <has> the same form [… ] and your boomerang <are> finish
3. 113161 Russian-German bilingual
           You have to fix the wood with the table and cut it <carefully> out
4. 143577 German monolingual
           <boring machine> (instead of *drill*; German: *Bohrmaschine*)
5. 143009 German monolingual
           <a thing to put some holes into wood>

Finally, note that the task did not trigger third person singular agreement, with which the majority of informants had difficulties in the other tasks we conducted.

### Scores in english with regard to scores in german and the heritage languages

Tables 2, 3 below show the 10 informants with the highest and lowest scores in English, listed from highest to lowest, along with relevant background variables and the language assessment scores in German and their heritage languages. The scores in the main panel were calculated with a mean value of 100 and a standard deviation of 20. As two waves of testing were conducted in the context of LiPS, two sets of scores are available. Here, we focus on the scores from the second wave.

**Table 2:** **Informants with highest attainment scores, including academic literacy scores for German/heritage languages**.

**ID**	**Group**	**Sex**	**OSG**	**HISCED**	**HISEI**	**School**	**LASG**	**LASHL**	**TSE**
143032	Ger.	f	ml	Level 6	88	Gym	148.57	ml	6.0
143387	Ger.	m	ml	Level 6	65	Gym	134.59	ml	6.0
133044	Viet.-Ger.	f	6	Level 6	43	Gym	110.79	88.8	6.0
143009	Ger.	m	ml	Level 6	51	Gym	128.16	ml	6.0
143411	Ger.	f	ml	Level 6	77	Gym	114.41	ml	5.5
143396	Ger.	m	ml	Level 6	71	Gym	169.26	ml	5.5
113183	Rus.-Ger.	f	n/a	n/a	n/a	Real	112.30	106.24	5.5
113090	Rus.-Ger.	f	6	Level 6	67	Gym	88.00	96.99	5.0
113186	Rus.-Ger.	f	3	Level 6	57	Gym	113.35	109.97	5.0
113193	Rus.-Ger.	m	0	Level 6	71	Gym	143.49	165.19	5.0

OSG, Onset German; HISCED, Highest educational background in informants' families; HISEI, Highest socioeconomic background of the informants' households; LASG, Language Assessment Score German; LASCHL, Language Assessment Score Heritage Language; TSE, Total Score English; ml, monolingual; Gym, Gymnasium; Real, Realschule.

**Table 3:** **Informants with lowest attainment scores, including academic literacy scores for German/heritage languages**.

**ID**	**Group**	**Sex**	**OSG**	**HISCED**	**HISEI**	**School**	**LASG**	**LASHL**	**TSE**
133098	Viet.-Ger.	f	3	Level 6	67	Gym	104.38	117.60	2,5
113177	Rus.-Ger.	f	3	Level 6	32	Gym	77.06	58.39	1.0
133130	Viet.-Ger.	m	>6	Level 6	49	Stadt	75.45	126.24	1.0
123240	Turk.-Ger.	f	0	Level 3	39	Stadt	98.58	102.59	1.0
123236	Turk.-Ger.	f	0	Level 2	43	Gym	n/a	n/a	1.0
113156	Rus.-Ger.	f	0	Level 2	30	Real	93.47	87.68	1.0
123163	Turk.-Ger.	f	0	n/a	n/a	Gesamt	99.79	123.19	1.0
123241	Turk.-Ger.	m	0	Level 3	49	Stadt	61.00	73.62	0.5
143565	Ger.	f	ml	Level 3	32	S/F	72.20	ml	0.0
143327	Ger.	f	ml	Level 3	33	S/F	101.52	ml	0.0

OSG, Onset German; HISCED, Highest educational background in informants' families; HISEI, Highest socioeconomic background of the informants' households; LASG, Language Assessment Score German; LASCHL, Language Assessment Score Heritage Language; TSE, Total Score English; ml, monolingual; Gym, Gymnasium; Stadt, Stadtteilschule; Real, Realschule; Gesamt, Gesamtschule; S/F, Sonderschule/Förderschule.

Again, there does not seem to be any effect attributable to either sex or age of onset regarding the scores in German and the heritage language. With the exception of informant 113090, informants with high scores in English also achieve high scores in German.

Of the 10 informants with the highest scores, five are bilinguals. Four of these belong to the Russian-German group. The remaining bilingual informant stems from the Vietnamese-German group, achieving the highest score of all bilinguals. This informant (133044) speaks primarily German at home. Her scores in German are higher than in Vietnamese. Her assessment scores for German, however, are lower than those of the monolingual German informants (except informant 143411). Further background variables reveal that informant 133044 is a special case, as she attended an international school in Vietnam where English was the first language that she formally acquired. This is one of the primary reasons for her high literacy score in English.

Three of the Russian-German bilinguals achieved high scores in their heritage language Russian. Informant 113090, a Russian-German bilingual attending the *Gymnasium*, shows low assessment scores in German and her heritage language, even though she achieved comparatively high literacy levels in the English Boomerang task. HISCED and HISEI levels are both high in her case.

At the lower end of the scoring spectrum for English (see Table 3), we also find lower literacy scores for German. Except for informant 113177, who shows lower scores in both background languages though better results for German, all of the informants show higher results in their heritage languages. Eight of the 10 informants are of multinational descent. The two informants with the lowest literacy scores represent the two German monolingual girls receiving specialized schooling for children with learning disabilities. Their results for English can be interpreted as a consequence of their learning disabilities. Four of the 10 informants with lower attainment levels in English are Turkish-German bilinguals, with *n* = 5 informants in our sub-sample being of Turkish descent.

We may suspect a correlation between literacy attainment in German and English. A Pearson correlation for our bilingual informants reveals that the raw scores for the English Boomerang task correlate at the 0.01 level with the scores in the German Boomerang task. There is no significant correlation between the scores for the heritage languages and English. These results are summarized in Table 4.

**Table 4:** **Pearson correlation scores**.

	**Score English**	**Score German**	**Score heritage languages**
Score English	1	0.580^**^	0.277
		0.000	0.113
Score German	0.580^**^	1	0.638^**^
	0.000		0.000
Score heritage languages	0.277	0.638^**^	1
	0.113	0.000	

** Correlation is significant at the 0.01 level (2-tailed); Listwise N = 34.

### Bilingual informants' heritage literacy assessment scores in relation to their academic achievement in english literacy

If we look only at our bilingual informants and rearrange our results according to the 10 informants with the highest literacy scores in their heritage languages, we find mixed results for their scores in English. If, however, we look at the 10 informants with the lowest assessment scores in their heritage language, this results in the picture shown in Table 5.

**Table 5:** **Bilingual informants with lowest academic literacy scores in their heritage languages (Second wave)**.

**ID**	**Group**	**Sex**	**OSG**	**HISCED**	**HISEI**	**School**	**LASG**	**LASHL**	**TSE**
113209	Russ.-Ger.	f	3	Level 3	53	n/a	91.38	93.58	4.0
133044	Viet.-Ger.	f	6	Level 6	43	Gym	110.79	88.80	6.0
113156	Russ.-Ger.	f	0	Level 2	30	Real	93.47	87.68	1.0
133088	Viet.-Ger.	m	3	n/a	30	Gym	95.20	83.04	2.5
133008	Viet.-Ger.	m	3	n/a	31	Real	77.25	80.16	2.5
133099	Viet.-Ger.	m	3	n/a	45	Gym	81.30	80.16	2.5
133150	Viet.-Ger.	m	3	n/a	34	Stadt	81.10	80.16	2.5
123241	Turk.-Ger.	m	0	Level 3	49	Stadt	61.00	73,62	0.5
133007	Viet.-Ger.	f	3	n/a	30	Gym	96.09	60.00	2.5
113177	Russ.-Ger.	f	3	Level 6	32	Gym	77.06	58.39	1.0

OSG, Onset German; HISCED, Highest educational background in informants' families; HISEI, Highest socioeconomic background of the informants' households; LASG, Language Assessment Score German; LASCHL, Language Assessment Score Heritage Language; TSE, Total Score English; Gym, Gymnasium; Stadt, Stadtteilschule; Real, Realschule; Gesamt, Gesamtschule.

Informants with comparatively lower language assessment scores in the heritage language versions of the Boomerang task reach comparatively low literacy scores in the English Boomerang task. The exception to this is informant 133044, who, as has already been established, acquired English as her first formal language and achieves a relatively high score in the German version of the Boomerang task. Informant 113209 is an interesting case, because she reaches an above average literacy score in English, but comparatively lower levels of literacy in German and her heritage language. In this case, however, the family has a comparatively high educational background. Moreover, in the first wave of testing, she obtained a relatively high literacy score in Russian.

### Summary of results

To sum up, it does seem to be the case that higher academic literacy scores in both German and the heritage language coincide with higher academic literacy scores in English. Whether or not literacy resources are accessible, however, seems to be dependent on language external factors. Moreover, comparatively higher academic literacy assessment scores in German seem to coincide more frequently with high academic literacy scores in English. Higher literacy scores in the informant's heritage language coinciding with comparatively lower attainment scores in German result in a lower academic literacy score in English. In general the lowest assessment scores in bilingual informants' heritage languages coincide with low academic literacy outcomes for English.

## Discussion

In this exploratory, qualitative analysis of our data set, we found no evidence for advantages of bilinguals regarding the production of written instructive texts in English, in comparison to their monolingual peers. Our results, however, show that bilingual informants with high assessment levels for academic literacy in both German and their heritage language are more likely to achieve better results in the production of academic English. In our view, this finding supports Cummins' Threshold Hypothesis as long as we view English literacy as a form of academic attainment. Moreover, informants with low literacy attainment levels in their heritage languages achieved comparatively lower scores for the task at hand.

Our results also show that there are individual thresholds, as some informants seem to be able to access their literacy resources on lower levels than others. The accessibility of these lower level resources seems to be dependent on socioeconomic background variables, as informants with low literacy scores in their background languages—though attaining high literacy scores in the English task—have a high socioeconomic and educational background.

Although we did not find evidence for advantages of bilingual informants regarding their literacy levels in English, it must be noted that there may be factors overriding our results. For example, 16-years-old students in Germany are likely to have encountered instructive text types in school and in their environment in German, but are much less likely to have encountered them in their heritage language, especially since the majority of our informants received no formal training in their home languages. Other overriding factors include the quality of English language education and students' motivation to learn English.

Even though our study is primarily concerned with literacy attainment, it would appear justified to take a brief look at the influencing factors identified in L3 acquisition studies. In this context, for example, typological proximity has been identified as an important factor (De Angelis, 2007, pp 22–31). In his *Typological Primacy Model* (2011), Rothman postulates that typological proximity is the strongest factor influencing syntactic transfer in L3 acquisition processes. Due to the close proximity of German and English, it appears possible that our informants rely more heavily on German than on their heritage languages in the production of English, even if their heritage language knowledge is comparatively high. This may even apply to informants whose heritage language is dominant, as is the case for some of the Russian-German informants. This claim seems to be supported by the fact that informants code-mix almost exclusively from German and that the majority of direct lexical transfer patterns that we can observe come from German. Another possible explanation for these observations is that transfer occurs from German because it is the L2, thus supporting the L2 status factor model initially put forth by Hammarberg (2001) and recently developed further by Bardel and Falk (2007) and Falk and Bardel (2011)^5^. According to the L2 status factor model, the L2 blocks out influence from L1, thus acting like a veil. Subsequently, we can make a strong case for a scenario in which high literacy scores in German are more likely to coincide with high literacy scores in English, not only due to the nature of the investigated task, but also due to the typologies of the languages involved.

It is important to note that we do not find any negative effects of high literacy scores in bilingual informants' heritage languages regarding their production of English academic texts. To the contrary, informants with high literacy assessment scores in both German *and* their heritage language are more likely to show higher literacy scores in English, though high assessment scores in German seems to be a precondition in this particular case. Moreover, informants with low literacy levels in their heritage languages have lower literacy scores in English. Informants' literacy levels in their heritage languages, therefore, contribute directly to their attainment of English literacy.

As already alluded to, it is difficult to compare informants' literacy skills in their heritage language to their literacy skills in German. Still, we would like to emphasize that due to the nature of bilingualism, comparing bilingual children's results in German or their heritage languages to monolingual children's results in German is also quite problematic. This follows from the fact that bilinguals cannot be viewed as a combination of two monolinguals, as established by Grosjean (1989).

Pursuing this line of thought further, we would also like to note that the comparison of bilingual and monolingual learners in the acquisition of English as an additional language poses similar difficulties. That is to say, advantages for bilinguals are more likely in domains where the same attainment levels have been reached for both their native languages. As children and adolescents who grow up in a monolingual environment are likely to have a more diverse knowledge of different registers than their bilingual peers, this may account for the high literacy scores that some of our monolingual informants achieve in English and disadvantage multilingual children in language assessment tasks for additional languages.

Another important factor to keep in mind with regard to our data is that there were 16 monolingual and 36 bilingual informants in total, and that the bilingual group was heterogeneous in terms of language background. Out of the bilingual informants, 11 were Vietnamese-German bilinguals and only 5 were Turkish-German bilinguals, while the others belonged to the Russian-German group. It is therefore difficult to draw conclusions concerning specific migration or language backgrounds.

## Concluding remarks

The exploratory study presented here is primarily intended as a trend study for further research. While we do find tendencies supporting Cummins' Threshold Hypothesis, the highly heterogeneous nature of the investigated data has to be kept in mind. Moreover, literacy and socioeconomic conditions do not cumulatively impact on the attainment of English as an additional language, at least not necessarily. Bilingual individuals achieving high literacy in German and their heritage languages also show high literacy achievement in English. While socioeconomic and educational background intensify these effects, informants with favorable socioeconomic preconditions are more likely to achieve high literacy levels. This means that they are able to access linguistic, or more specifically literacy, resources at a lower threshold.

At the same time, bilinguals of lower socioeconomic backgrounds can realize high literacy scores in English, if they achieve higher assessment scores in German *and* their heritage languages. In other words, they are able to access literacy resources at a higher threshold, but once they do, they perform at a high level. In our task, high literacy achievement in German coincided more often with high literacy achievement in English than high literacy achievement in informants' heritage languages. We argue that this is due to both the typologies of the languages involved and the task at hand, which tested for academic language and the production of instructive text types. More importantly, low literacy achievement in informants' heritage languages resulted in lower literacy achievement in English. A low level of literacy achievement in these languages can therefore be viewed as detrimental to literacy attainment in the acquisition processes of additional languages. Our results are therefore in line with findings by Swain et al. (1990) who suggest that L1 literacy is a key factor for positive effects in the attainment of additional languages in migrant contexts.

### Conflict of interest statement

The authors declare that the research was conducted in the absence of any commercial or financial relationships that could be construed as a potential conflict of interest.

## Footnotes

^1^*Fast Catch Bumerang* is the German trademark of the instrument.

^2^Some of the informants refused to participate in the Boomerang task, especially in the German-Vietnamese group. The reasons for this were diverse. Some informants thought that the task was too difficult, others simply did not want to do the task again, as they had already completed it in German and their heritage language.

^3^The ISCED (International Standard Classification of Education) ranges from Level 1 to 6 and measures the educational background of informants' families. HISCED refers to the highest ISCED in informants' families (cf. Ehmke and Thilo, 2005).

^4^The ISEI (International Socio-Economic Index of Occupational Status) ranges from 16 to 90 and is based the occupation and related income of informants' parents. HISEI refers to the highest ISEI in the family (cf. Ehmke and Thilo, 2005).

^5^We would like to point out that both Rothman as well as Bardel and Falk define a true L2 as a non-native language (Bardel and Falk, 2007, p. 460), and that our findings would be more adequately viewed as L2 rather than L3 acquisition in the given context. We would also like to emphasize that the distinction between L2 and L3 acquisition is extremely difficult to establish for our groups of informants.
