# Supplementary material for: Double threshold in bi- and multilingual contexts: preconditions for higher academic attainment in English as an additional language
Source: Front Psychol. 2014 Jun 5;5:546. doi: 10.3389/fpsyg.2014.00546 (PMC4046175; doi:10.3389/fpsyg.2014.00546)
Supplement: Supplementary file 1 [file DataSheet1.DOCX]

Appendix

| **ID** | **Group** | **TSE** | **SEX** | **OSG** | **HISCED** | **HISEI** | **School Form** |
| --- | --- | --- | --- | --- | --- | --- | --- |
| 143032 | Ger. | 6.0 | f | ml | Level 6 | 88 | Gymnasium |
| 143387 | Ger. | 6.0 | m | ml | Level 6 | 65 | Gymnasium |
| 133044 | Viet.-Ger. | 6.0 | f | 6 | Level 6 | 43 | Gymnasium |
| 143009 | Ger. | 6.0 | m | ml | Level 6 | 51 | Gymnasium |
| 143411 | Ger. | 5.5 | f | ml | Level 6 | 77 | Gymnasium |
| 143396 | Ger. | 5.5 | m | ml | Level 6 | 71 | Gymnasium |
| 113183 | Rus.-Ger. | 5.5 | f | n/a | n/a | n/a | Realschule |
| 113090 | Rus.-Ger. | 5.0 | f | 6 | Level 6 | 67 | Gymnasium |
| 113186 | Rus.-Ger. | 5.0 | f | 3 | Level 6 | 57 | Gymnasium |
| 113193 | Rus.-Ger. | 5.0 | m | 0 | Level 6 | 71 | Gymnasium |
| 133149 | Viet.-Ger. | 4.5 | f | 4 | Level 5 | 43 | Gymnasium |
| 113213 | Rus.-Ger. | 4.5 | f | 3 | Level 6 | 53 | Gymnasium |
| 143458 | Ger. | 4.0 | m | ml | Level 5 | 53 | Gymnasium |
| 113162 | Rus.-Ger. | 4.0 | f | > 6 | Level 4 | 34 | Gymnasium |
| 133053 | Viet.-Ger. | 4.0 | m | 3 | n/a | 16 | Gymnasium |
| 113153 | Rus.-Ger. | 4.0 | m | 4 | Level 6 | 71 | Gymnasium |
| 143403 | Ger. | 4.0 | m | ml | n/a | n/a | n/a |
| 113066 | Rus.-Ger. | 4.0 | f | 3 | Level 6 | 30 | Gymnasium |
| 113209 | Rus.-Ger. | 4.0 | f | 3 | Level 3 | 53 | n/a |
| 143558 | Ger. | 4.0 | m | ml | Level 6 | 69 | Gymnasium |
| 143581 | Ger. | 3.5 | f | ml | Level 4 | 30 | Stadtteilschule |
| 113184 | Rus.-Ger. | 3.5 | f | 0 | n/a | 49 | Gymnasium |
| 133069 | Viet.-Ger. | 3.5 | f | 3 | n/a | 49 | Gymnasium |
| 113161 | Rus.-Ger. | 3.0 | f | > 6 | Level 6 | 88 | Gymnasium |
| 113180 | Rus.-Ger. | 3.0 | f | n/a | n/a | n/a | Gymnasium |
| 123237 | Turk.-Ger. | 2.5 | m | 0 | Level 4 | 50 | Gymnasium |
| 113168 | Rus.-Ger. | 2.5 | m | 4 | Level 6 | n/a | Gymnasium |
| 113089 | Rus.-Ger. | 2.5 | f | 3 | Level 5 | 51 | Stadtteilschule |
| 143131 | Ger. | 2.5 | m | ml | Level 4 | 45 | Gymnasium |
| 133007 | Viet.-Ger. | 2.5 | f | 3 | n/a | 30 | Gymnasium |
| 113026 | Rus.-Ger. | 2.5 | f | > 6 | Level 5 | 45 | Gymnasium |
| 113006 | Rus.-Ger. | 2.5 | m | > 6 | Level 6 | 56 | Gymnasium |
| 143577 | Ger. | 2.5 | n/a | ml | n/a | n/a | Stadtteilschule |
| 143400 | Ger. | 2.5 | m | ml | Level 3 | 56 | Realschule |
| 143034 | Ger. | 2.5 | m | ml | Level 6 | 30 | Gesamtschule |
| 133088 | Viet.-Ger. | 2.5 | m | 3 | n/a | 30 | Gymnasium |
| 133150 | Viet.-Ger. | 2.5 | m | 3 | n/a | 34 | Stadtteilschule |
| 113194 | Rus.-Ger. | 2.5 | f | 6 | Level 6 | 34 | Gymnasium |
| 143113 | Ger. | 2.5 | m | ml | Level 3 | 56 | Gesamtschule |
| 133099 | Viet.-Ger. | 2.5 | m | 3 | n/a | 45 | Gymnasium |
| 113212 | Rus.-Ger. | 2.5 | f | 6 | Level 6 | 30 | Realschule |
| 133008 | Viet.-Ger. | 2.5 | m | 3 | n/a | 31 | Realschule |
| 133098 | Viet.-Ger. | 2.5 | f | 3 | Level 6 | 67 | Gymnasium |
| 113177 | Rus.-Ger. | 1.0 | f | 3 | Level 6 | 32 | Gymnasium |
| 133130 | Viet.-Ger. | 1.0 | m | > 6 | Level 6 | 49 | Stadtteilschule |
| 123240 | Turk.-Ger. | 1.0 | f | 0 | Level 3 | 39 | Stadtteilschule |
| 123236 | Turk.-Ger. | 1.0 | f | 0 | Level 2 | 43 | Gymnasium |
| 113156 | Rus.-Ger. | 1.0 | f | 0 | Level 2 | 30 | Realschule |
| 123163 | Turk.-Ger. | 1.0 | f | 0 | n/a | n/a | Gesamtschule |
| 123241 | Turk.-Ger. | 0.5 | m | 0 | Level 3 | 49 | Stadtteilschule |
| 143565 | Ger. | 0.0 | f | ml | Level 3 | 32 | Sonderschule/Förderschule |
| 143327 | Ger. | 0.0 | f | ml | Level 3 | 33 | Sonderschule/Förderschule |

*Table 2: Informants according to attainment score in English. (TSE = Total Score English, OSG = Onset German, HISCED = highest educational background in informants’ families, HISEI = highest socioeconomic background of the informants’ households, ml = monolingual).*
